# Present and future pharmacotherapeutic agents in heart failure: an evolving paradigm

**DOI:** 10.1111/bph.13480

**Published:** 2016-05-06

**Authors:** Brodie L Loudon, Hannah Noordali, Nicholas D Gollop, Michael P Frenneaux, Melanie Madhani

**Affiliations:** ^1^ Faculty of Medicine and Health SciencesUniversity of East AngliaNorwichUK; ^2^ Institute of Cardiovascular SciencesUniversity of BirminghamBirminghamUK

## Abstract

Many conditions culminate in heart failure (HF), a multi‐organ systemic syndrome with an intrinsically poor prognosis. Pharmacotherapeutic agents that correct neurohormonal dysregulation and haemodynamic instability have occupied the forefront of developments within the treatment of HF in the past. Indeed, multiple trials aimed to validate these agents in the 1980s and early 1990s, resulting in a large and robust evidence‐base supporting their use clinically. An established treatment paradigm now exists for the treatment of HF with reduced ejection fraction (HFrEF), but there have been very few notable developments in recent years. HF remains a significant health concern with an increasing incidence as the population ages. We may indeed be entering the surgical era for HF treatment, but these therapies remain expensive and inaccessible to many. Newer pharmacotherapeutic agents are slowly emerging, many targeting alternative therapeutic pathways, but with mixed results. Metabolic modulation and manipulation of the nitrate/nitrite/nitric oxide pathway have shown promise and could provide the answers to fill the therapeutic gap between medical interventions and surgery, but further definitive trials are warranted. We review the significant evidence base behind the current medical treatments for HFrEF, the physiology of metabolic impairment in HF, and discuss two promising novel agents, perhexiline and nitrite.

**Abbreviations**
ANT: adenine nucleotide translocase
ARB: angiotensin receptor blocker
BEAUTIFUL: morBidity‐mortality EvAlUaTion of the I*f* inhibitor ivabradine in patients with coronary disease and left ventricULar dysfunction trial
BNP: brain natriuretic peptide
CHARM: Candesartan in Heart failure ‐ Assessment of moRtality and Morbidity
CK: creatine kinase
COPERNICUS: CarvedilOl ProspEctive RaNdomIzed CUmulative Survival Study
CPT: carnitine palmitoyltransferase
ESC: European Society of Cardiology
FA: fatty acid
FADH2: flavin adenine dinucleotide
HF: heart failure
HFpEF: heart failure and preserved ejection fraction
HFrEF: heart failure and reduced ejection fraction
ISDN: isosorbide dinitrate
LVEF: left ventricular ejection fraction
MERIT‐HF: MEtoprolol cr/xl Randomised Intervention Trial in congestive Heart Failure
NYHA: New York Heart Association
PDH: pyruvate dehydrogenase
PCr: phosphocreatine
P_i_: inorganic phosphate
PPP: pentose phosphate pathway
RAAS: renin–angiotensin–aldosterone system
RCT: randomized controlled trial
SENIORS: Study of the Effects of Nebivolol Intervention on Outcomes and Rehospitalization in Seniors with heart failure
SHIFT: Systolic Heart failure treatment with the If inhibitor ivabradine Trial
TCA: tricarboxylic acid
UCP: uncoupling protein
V‐HeFT: vasodilator heart failure trial

## Tables of Links

**Table:** 

**TARGETS**
**GPCRs** ^*a*^
AT_1_ receptor
β‐adrenoceptors
**Nuclear hormone receptors** ^*b*^
Mineralocorticoid receptor
PPARα
**Enzymes** ^*c*^
ACE
Aldehyde dehydrogenase 2
Neprilysin
NOS
PDE5
Soluble guanylate cyclase
**Transporters** ^*d*^
Adenine nucleotide translocase (ANT)
ATP synthase
GLUT1
GLUT4
Uncoupling protein (UCP)

**LIGANDS**		
Acetyl CoA	cGMP	Metoprolol
ADP	C‐type natriuretic peptide	Nebivolol
Aldosterone	Digoxin	Nitric oxide (NO)
Angiotensin II	Enalapril	Nitroglycerin
ATP	Eplerenone	Omecamtiv mecarbil
Atrial natriuretic peptide (ANP)	Hydralazine	Prazosin
Bisoprolol	Insulin	Pyruvate
BNP	Isosorbide dinitrate	Ranolazine
Bradykinin	Isosorbide mononitrate	Spironolactone
cAMP	Ivabradine	TNF‐α
Candesartan	Lactate	Valsartan
Captopril	L‐arginine	Verapamil
Carvedilol	LCZ696 (sacubitril)	

These Tables list key protein targets and ligands in this article which are hyperlinked to corresponding entries in http://www.guidetopharmacology.org, the common portal for data from the IUPHAR/BPS Guide to PHARMACOLOGY (Pawson *et al*., 2014) and are permanently archived in the Concise Guide to PHARMACOLOGY 2015/16 (^*a,b,c,d*^Alexander *et al*., 2015a, 2015b, 2015c, 2015d).

## Introduction

Chronic heart failure (HF) is a complex, systemic, multi‐organ syndrome, which represents a common endpoint for many cardiac conditions (Follath *et al*., 1998). Traditionally, it is characterized by haemodynamic impairment and progressive neurohormonal dysregulation, with increased sympathetic activation, elevated peripheral vascular resistance and cardiac remodelling (Braunwald and Bristow, 2000). More recently, metabolic impairment (Neubauer, 2007) and activation of inflammatory responses (Rauchhaus *et al*., 2000) have been increasingly recognized. At a population level, HF affects around 900 000 Britons, half of whom die within 5 years of diagnosis (Hobbs *et al*., 2007). At current hospitalization rates, 70% of which involve patients aged >65 years, HF costs the UK 2% of the total annual National Health Service budget (Basu *et al*., 2008), and this is set to increase further as the population ages.

Pharmacotherapeutic agents geared towards correcting neurohormonal dysregulation have occupied the forefront of developments within the treatment of HF in the past, resulting in a large and robust evidence‐base supporting their use clinically. This evidence underpins the European Society of Cardiology (ESC) Guidelines for the Diagnosis and Treatment of Acute and Chronic Heart Failure, most recently updated in 2012 (McMurray *et al*., 2012). The pace and success of development of novel pharmacotherapeutic agents over the last 20 years has been disappointing. However, new agents are slowly emerging. LCZ696 (a combination therapy of an angiotensin receptor blocker and a neprilysin inhibitor) demonstrated superiority over angiotensin converting enzyme (ACE) inhibitors in 8399 patients with HF and reduced ejection function (HFrEF), with a reduction in the risk of all‐cause mortality of 12.6% (95% CI 7–18%; *P* < 0.0001) (Packer *et al*., 2015). Inflammatory mediators such as the key cytokine TNF‐α were also identified as targets for novel therapies in patients with HF; however, human trials have shown disappointing results (Chung *et al*., 2003; Mann *et al*., 2004; Torre‐Amione *et al*., 2008). Despite this, a significant advancement in our understanding of inflammation in HF has been made and has generated further research in this field (Heymans *et al*., 2009). Investigation into cardiac myosin activators [such as omecamtiv mecarbil, which has been shown to improve systolic function and is well tolerated (Greenberg *et al*., 2015)], phosphodiesterase inhibition, manipulation of the nitrate/nitrite/NO pathway, metabolic modulation, stem cell therapy and gene therapy is currently underway (Shah and Mann, 2011; Tilemann *et al*., 2012).

Up to 50% of patients with HF have a preserved ejection fraction of ≥50% (HFpEF). The therapeutic paradigm that has been successful in HFrEF has failed in this syndrome (Yusuf *et al*., 2003; Cleland *et al*., 2006; Massie *et al*., 2008; Pitt *et al*., 2014). Therefore, effective therapies are urgently needed. With the rapid advancements in the development of implantable cardiac devices such as left ventricular assist devices, we may be entering the surgical era of HF treatment. These interventions, despite their clearly beneficial use in patients awaiting cardiac transplantation or as an alternative destination therapy, remain expensive and lead to device‐related complications. As such, surgery is reserved for patients with end‐stage HF (Mancini and Colombo, 2015).

**Figure 1:**
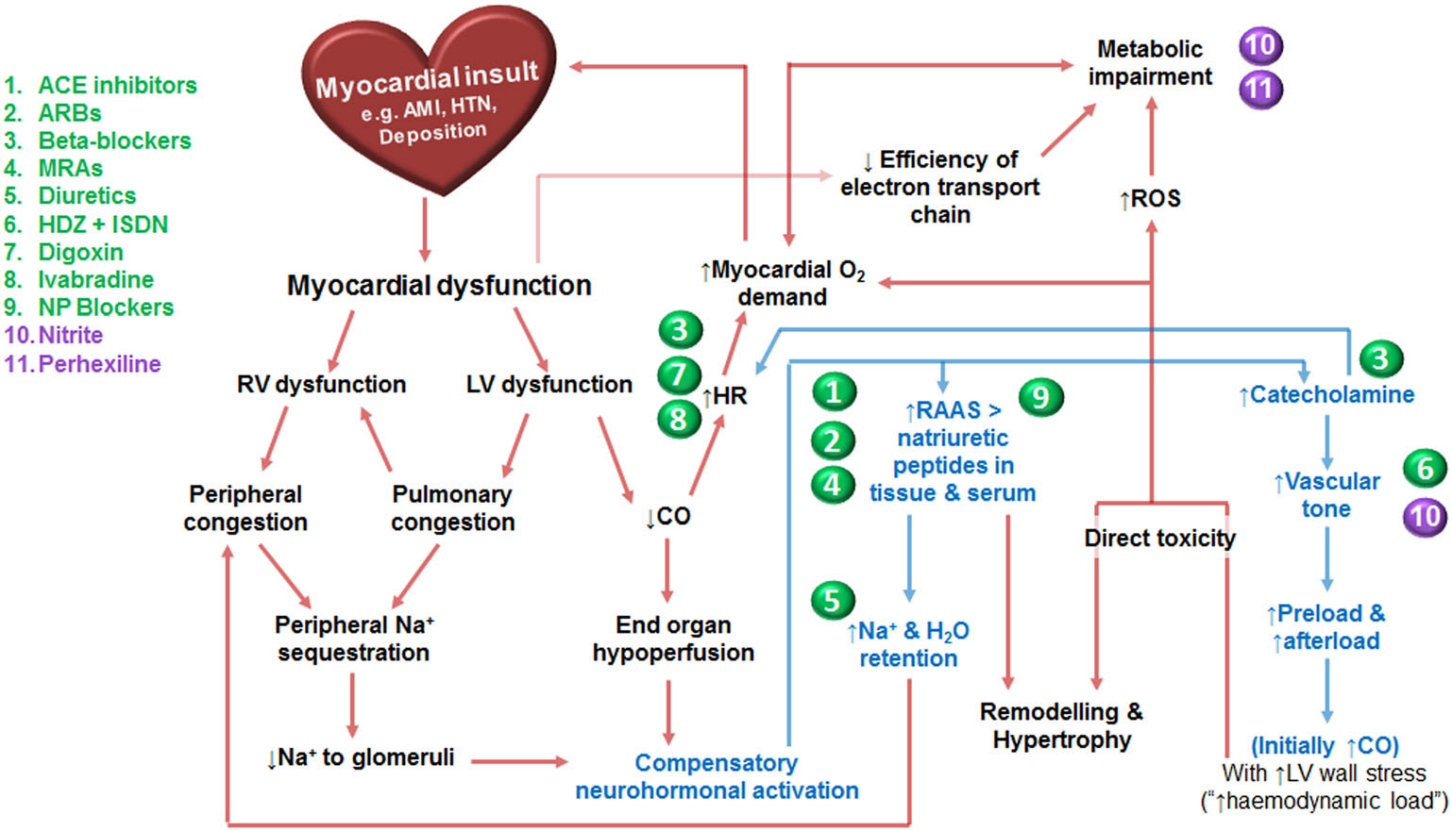
Overview of compensatory mechanisms in HF and complementary pharmacotherapeutic agents. Compensatory mechanisms and key pathophysiological changes take place in HF. Multiple pharmacotherapeutic agents have been developed to target these pathways and improve disease burden in HFrEF. ACE, angiotensin converting enzyme; AMI, acute myocardial infarction; ARBs, angiotensin receptor blockers; CO, cardiac output; H_2_O, water; HDZ, hydralazine; HR, heart rate; HTN, hypertension; ISDN, isosorbide dinitrate; LV, left ventricle; MRAs, mineralocorticoid receptor antagonists; Na, sodium; NP, neprilysin; RAAS, renin–angiotensin–aldosterone system; ROS, reactive oxygen species; RV, right ventricle.

We suggest that targeting alternative pathways other than those used by the well‐established HFrEF pharmacological agents will prove beneficial in the advancement of pharmacotherapy in HF (Figure 1). Metabolic modulation has shown a promising and favourable effect profile in Phase 2 randomized controlled trials (RCTs) performed by our group in patients with HFrEF (Lee *et al*., 2005) and hypertrophic cardiomyopathy (Abozguia *et al*., 2010). However, to date, there have been no Phase 3 trials. A meta‐analysis assessing the addition of trimetazidine to conventional therapy in patients with HFrEF demonstrated a reduction in hospitalization for cardiac causes (RR: 0.43, *P* = 0.03) and improved symptomatology and parameters of cardiac functioning, but had no effect on all‐cause mortality (Zhang *et al*., 2012). Our group and others have also investigated nitrite, which has been shown to be a hypoxic‐dependent vasodilator, significantly improving cardiac haemodynamics without causing symptomatic hypotension (Maher *et al*., 2008). Nitrite has also been shown to exhibit additional benefits of metabolic modulation within skeletal muscle (Bailey *et al.*, 2009), which may translate to cardiac muscle. Herein, we will review the significant evidence base behind the current medical treatments for HFrEF, the physiology of metabolic impairment in HF and discuss two promising novel agents, perhexiline and nitrite.

## ESC guidelines

The ESC guidelines recommend approved agents for use in HFrEF based on robust clinical evidence (Table 1). Pharmacotherapeutic agents with class I (strong evidence of benefit) recommendations include β‐blockers and ACE inhibitors [or angiotensin receptor blockers (ARBs) when ACE inhibitors are not tolerated] for all patients with HFrEF in the first instance, regardless of symptomatology. Additional agents including hydralazine and isosorbide dinitrate [in African‐American patients with New York Heart Association (NYHA) class III–IV on an ACE inhibitor and β‐blocker] and mineralocorticoid receptor antagonists [NYHA class II–IV with left ventricular ejection fraction (LVEF) ≤35%] are indicated as necessitated by ongoing NYHA class II–IV symptoms. These medications are supported by class A evidence (data from multiple RCTs). Recommendations in favour of treatment being beneficial (class IIb) have also been made by the ESC for the use of digoxin and ivabradine (if HR ≥70 bpm) in HFrEF with sinus rhythm, unless contraindicated, to decrease hospitalizations (McMurray *et al*., 2012).

**Table 1:** Pivotal clinical trials for pharmacotherapeutic agents approved for the treatment of HFrEF

HFrEF agent	Example	Relevant trials	Number of patients	Relative risk		Reference
				All‐cause mortality (95% CI)	Heart failure hospitalizations (95% CI)	
Angiotensin converting enzyme (ACE) inhibitors	Enalapril	CONSENSUS trial	2289	0.73 (0.63–0.84, *P* < 0.00002)	0.73 (N/A, *P* = 0.0001)	CONSENSUS Trial Study Group, 1987
		SOLVD trial	2569	0.84 (0.74–0.95, *P* = 0.0036)	0.74 (0.66–0.72, *P* < 0.0001)^ǂ^	The SOLVD Investigators, 1992
	Captopril	SAVE trial	2231	0.81 (0.68–0.97, *P* = 0.019)	0.78 (0.63–0.96, *P* = 0.019)	Pfeffer *et al*., 1992
	Ramipril	AIRE trial	2006	0.73 (0.60–0.89, *P* = 0.002)	N/A	The AIRE Study Group, 1993
Angiotensin receptor blockers (ARBs)	Candesartan	CHARM‐alternative trial	2028	0·80 (0·66–0·96, *P* = 0.02)	0·61 (0·51–0·73, *P* < 0.0001)	Granger *et al*., 2003
	Valsartan	Val‐HeFT trial (added to ACE‐Is)	5010	1.02 (0.88–1.1, *P* = 0.80)^§^	0.87 (0.77–0.97, *P* = 0.009)^*^ ^,^ ^ǂ^	Cohn and Togononi, 2001
		VALIANT trial (non‐inferiority)	14 703	1.00 (0.90–1.11, *P* = 0.98)^*^ ^,^ ^¥^	0.97 (0.90–1.05, *P* = 0.51)^ǂ^ ^,^ ^¥^	Pfeffer *et al*., 2003
Mineralocorticoid receptor antagonists	Spironolactone	RALES trial	1663	0.70 (0.60–0.82, *P* < 0.001)	0.65 (0.54–0.77, *P* < 0.001)	Pitt *et al*., 1999
	Eplerenone	EMPHASIS‐HF trial	2737	0.76 (0.62–0.93, *P* = 0.008)	0.58 (0.47–0.70, *P* < 0.001)	Zannad *et al*., 2011
β‐Blockers	Bisoprolol	CIBIS‐II trial	2647	0.66 (0.54–0.81, *P* < 0.0001)	0·64 (0·53–0·79, *P* = 0.001)	CIBIS‐II Study Group, 1999
	Metoprolol	MERIT‐HF trial	3991	0.66 (0.53–0.81, *P* = 0.0062)	0.65 (N/A)	MERIT‐HF Study Group, 1999
	Carvedilol	COPERNICUS trial	2289	0.65 (0.52–0.81, *P* = 0.00013)	0.72 (N/A, *P* = 0.0001)	Packer *et al*., 2002
	Nebivolol	SENIORS trial	2128	0.88 (0.71–1.08, *P* = 0.21)	0.86 (0.74–0.99, *P* = 0.039)^ǂ^	Flather *et al*., 2005
Hydralazine and isosorbide dinitrate	Hydralazine and ISDN	V‐HeFT I	642	0.66 (0.46–0.96, *P* < 0.028)	N/A	Cohn *et al*., 1986
		A‐HeFT	1050	0.57 (N/A, *P* = 0.01)	0.77 (N/A, *P* = 0.001)	Taylor *et al*., 2004
Cardiac glycosides	Digoxin	Digitalis Investigation Group trial	6800	0.99 (0.91–1.07, *P* = 0.80)	0.72 (0.66–0.79, *P* < 0.001)	The Digitalis Investigation Group, 1997
Ivabradine	Ivabradine	The BEAUTIFUL trial	10 917	1·04 (0·92–1·16, *P* = 0.55)	0·99 (0·86–1·13, *P* = 0.85)	Fox *et al*., 2008
		The SHIFT trial	6558	0·90 (0·80–1·02, *P* = 0.092)	0.74 (0.66–0.83, *P* < 0.0001)	Swedberg *et al*., 2010

aire, the Acute Infarction Ramipril Efficacy; concensus, the Cooperative North Scandinavian Enalapril Survival Study; EMPHASIS‐HF, the Eplerenone in Mild Patients Hospitalization and Survival Study in Heart Failure; HFrEF, Heart Failure with Reduced Ejection Fraction; N/A, Not Available; RALES, Randomized Aldactone Evaluation Study; SAVE, Survival and Ventricular Enlargement; SOLVD, Studies of Left Ventricular Dysfunction; VALIANT, Valsartan in Acute Myocardial Infarction; Val‐HeFT, Valsartan Heart Failure Trial.

^ǂ^ Includes death and hospitalization.

^*^ Confidence interval of 97.5%.

^§^ Confidence interval of 98%

^¥^ Compared with the Captopril treatment group.

## Evidence‐based pharmacological agents

### ACE inhibitors

Diminishing sodium delivery and arterial pressure to the renal glomeruli in patients with HF activates the renin–angiotensin–aldosterone system (RAAS), which is further augmented by sympathetic activation. This compensatory mechanism causes potent vasoconstriction (with an increase in total peripheral resistance and therefore afterload) and salt and water retention to increase circulatory volume (Furberg and Yusuf, 1985). ACE is a common link between the RAAS and kallikrein–kinin pathways, converting angiotensin I to the salt‐retentive, vasoconstrictive and hypertrophic angiotensin II, and degrading the vasodilatory and salt‐wasting bradykinin (Brown and Vaughan, 1998). It is now recognized that RAAS activation incorporates a tissue component (particularly cardiac, vascular and renal) that is independent from the systemic endocrine effects (Dzau and Re, 1994). In the compensated state of HF, plasma renin and ACE levels have been shown to fall in a trend towards normal (Dzau and Hirsch, 1990). Despite this trend, elevations in tissue RAAS persist, indicating its critical importance in cardiovascular disease states. Components of the RAAS at a tissue level act in a paracrine or autocrine manner to produce many tissue‐specific effects, including increased vascular tone and cardiac remodelling and fibrosis (Paul *et al*., 2006). Indeed, amelioration of chronic tissue RAAS activation is probably more important in the long‐term benefits of neurohormonal blockade than the haemodynamic effects of systemic RAAS blockade (Dzau and Hirsch, 1990).

Several influential RCTs investigating ACE inhibitors in HFrEF demonstrated reductions in mortality in the enalapril treatment groups compared with placebo of up to 31% (*P* = 0.001) (The CONSENSUS Trial Study Group, 1987; The SOLVD Investigators, 1992). Similar mortality benefits were later demonstrated for captopril and ramipril in the Survival And Ventricular Enlargement Trial and The Acute Infarction Ramipril Efficacy Study trials in 1992 and 1993, respectively (Pfeffer *et al*., 1992; The AIRE Study Group, 1993). In 1992, the Studies of Left Ventricular Dysfunction Prevention trial further demonstrated reduced HF incidence and cardiovascular mortality with enalapril, in patients with asymptomatic HFrEF (Cleland *et al*., 2006).

### Angiotensin receptor blockers

Angiotensin receptor blockers have similar therapeutic applications to ACE inhibitors in the setting of HF. They are useful as a monotherapy, provide an alternative option for individuals who are intolerant of ACE inhibitors (due to cough or angioedema) and may offer an augmented clinical effect in combination with ACE inhibitors (likely from a more extensive blockade of the RAAS axis). By competitively inhibiting angiotensin II at the angiotensin II type 1 (AT_1_) receptor directly, ARBs do not cause an elevation in bradykinin nor the attendant side effect profile. The most compelling data for ARBs versus placebo was presented by the Candesartan in Heart Failure ‐ Assessment of Mortality and Morbidity (CHARM) Program trials, the Valsartan Heart Failure Trial in 2001 and the Valsartan in Acute Myocardial Infarction trial in 2003. The CHARM Program trials demonstrated a significant reduction in cardiovascular death or hospital admission for HF in the candesartan group compared with placebo of 30% (95% CI 19–40%; *P* < 0.0001) in 2028 patients with symptomatic HFrEF who were not receiving ACE inhibitors due to previous intolerance (Granger *et al*., 2003; McMurray *et al*., 2003). The Valsartan Heart Failure Trial demonstrated a 24% (13.8% vs. 18.2%, *P* < 0.001) reduction in hospitalizations for worsening HF in the treatment group compared with placebo. However, 93% of study patients were also treated with ACE inhibitors, which is probably responsible for the lack of observed effect on mortality (Cohn and Tognoni, 2001). The Valsartan in Acute Myocardial Infarction trial was a three‐arm RCT of 14 808 patients comparing titrated valsartan, captopril and valsartan/captopril combination therapy in patients within 0.5–10 days of an acute myocardial infarction complicated by HFrEF and demonstrated non‐inferiority of valsartan to captopril (Pfeffer *et al*., 2003).

### Mineralocorticoid receptor antagonists

Direct blockade of aldosterone at the distal nephron promotes fluid loss and retention of potassium ions (otherwise lost as a result of the action of loop diuretics) and further amelioration of systemic and chronic tissue RAAS activation. The two main trials to investigate the role of aldosterone antagonists in patients with HFrEF were the Randomized Aldactone Evaluation Study 1996 and the Eplerenone in Mild Patients Hospitalization and Survival Study in Heart Failure 2011 studies. These RCTs compared spironolactone versus placebo in 1663 NYHA III–IV patients and eplerenone versus placebo in 2737 NYHA II patients, respectively on standard medical therapy. They demonstrated a 30% reduction in mortality in the spironolactone arm versus placebo at 24 months (95%CI 18–40%; *P* < 0.001) (Pitt *et al*., 1999) and a risk reduction of 37% in composite cardiovascular death and hospitalization for HF in the eplerenone group versus placebo at 21 months (95%CI 26–46%; *P* < 0.001) (Zannad *et al*., 2011) respectively. A further analysis of 261 patients from the Randomized Aldactone Evaluation Study revealed that high baseline levels of biomarkers of cardiac fibrosis (secondary to higher tissue RAAS activity) correlated with poorer prognosis, and the observed benefit from spironolactone was greater in such patients (Zannad *et al*., 2000).

### β‐blockers

Sympathetic neural activation in cardiac failure is a key compensatory mechanism to counteract falling cardiac output and end‐organ hypoperfusion. Chronically, however, elevated catecholamines are deleterious, with pro‐arrhythmogenic effects [with the attendant decrease in coronary blood flow and increased myocardial O_2_ demand (Rona, 1985)] and direct cardiotoxic effects via cAMP‐mediated calcium overload, particularly influx through the verapamil‐sensitive calcium channel, which is pro‐apoptotic (Mann *et al*., 1992). These effects are ameliorated by β‐Adrenoceptors antagonists, a heterogeneous group of pharmacotherapeutic agents. These agents vary greatly in their selectivity for the adrenoceptor subtypes and may possess ancillary properties (e.g. the antioxidant and anti‐inflammatory properties of carvedilol that are theoretically of great importance in HF); however, their impact on mortality appears to be a class‐wide effect (Chatterjee *et al*., 2013).

The four cornerstone trials assessing β‐blockers in HF were the The Cardiac Insufficiency Bisoprolol Study II (CIBIS‐II; 1999), Metoprolol cr/xl Randomized Intervention Trial in Congestive Heart Failure (MERIT‐HF; 1999), Carvedilol Prospective Randomized Cumulative Survival Study (COPERNICUS; 2002) and the Study of the Effects of Nebivolol Intervention on Outcomes and Rehospitalization in Seniors with heart failure (SENIORS; 2005) studies. The MERIT‐HF and CIBIS‐II trials in 1991 assessed controlled release metoprolol versus placebo in 3991 HFrEF patients and bisoprolol versus placebo in 2647 HFrEF patients, respectively (MERIT‐HF; The CIBIS‐II Study Group, 1999). Both trials demonstrated an impressive 34% reduction in mortality (95% CI 19–46%, *P* < 0.0001 and 95% CI 19–47%, *P* = 0.0062 respectively) in the treatment arm compared with placebo. The COPERNICUS trial assessed carvedilol versus placebo in 2289 patients with severe HF and LVEF ≤25% and demonstrated a 35% (95% CI 19–48%, *P* = 0.0014) reduction in mortality in the treatment group compared with placebo (Packer *et al*., 2002). A significant mortality benefit in 2128 patients >70 years of age was shown in the SENIORS trial in 2005, the first trial to specifically target HFrEF patients of advanced age. In the nebivolol treatment arm compared with placebo, there was a reduction in the composite primary outcome of all‐cause mortality and cardiovascular hospitalizations of 14% (95%CI 1–26%, *P* = 0.039) (Flather *et al*., 2005). This improvement in the composite outcome was driven mainly by a reduction in cardiovascular hospitalizations, with no effect on all‐cause mortality.

### Diuretics

The careful balance between fluid retention following chronic neurohormonal activation and compensatory natriuretic mechanisms is precarious and may be affected by the slightest changes in homeostasis. Fluid overload causes pulmonary congestion (in left heart failure) and gastrointestinal, hepatic and peripheral congestion (in right heart failure). This leads to the often debilitating and unacceptable symptomatology of the HF syndrome. Indeed, the evidence for the use of diuretics in HF is geared for the treatment of these symptoms and is experience‐based, with a paucity of robust clinical data in relation to improvements in disease mortality (Faris *et al*., 2002). Loop diuretics are the most efficacious and potent in alleviating symptoms, both in acute decompensation and chronic disease, but as with all diuretics, they must be carefully titrated against hypokalaemia (excluding potassium sparing), symptomatic hypotension and renal decline (a significant cause of mortality in patients who die from worsening HF (Sarraf *et al*., 2009).

### Hydralazine and isosorbide dinitrate

The effect of reducing both resistance vessel and venous tone on the failing heart via the simultaneous use of the arterial vasodilator hydralazine and the arteriolar and venodilator isosorbide dinitrate (ISDN) was assessed in the Vasodilator Heart Failure trials (V‐HeFT I/II) and the African‐American Heart Failure Trial in 1986, 1991 and 2004 respectively. The V‐HeFT I trial compared hydralazine and ISDN versus prazosin with placebo in 642 men with impaired systolic function and demonstrated a 34% relative risk reduction at 2 years (*P* < 0.028) (Cohn *et al*., 1986). V‐HeFT II was undertaken in 1991 to compare this combination with and without enalapril, with significant mortality benefit seen in the enalapril arm (Cohn *et al*., 1991). Hydralazine and ISDN in combination was also shown to reduce nitrate tolerance by previously unknown mechanisms. Organic nitrates activate NADPH oxidase, which results in deleterious ROS generation, partially contributing to nitrate tolerance via endothelial dysfunction (uncoupling of NOS) and inhibition of mitochondrial aldehyde dehydrogenase 2 (responsible for a large part of organic nitrate enzymatic bioactivation to NO) (Munzel *et al*., 2005). It seems fortuitous that hydralazine was used in addition to organic nitrates, as evidence later emerged that it prevents NADPH oxidase activation, reducing nitrate toxicity, tolerance and free radical production (Munzel *et al*., 1996). Given the mortality benefit, hydralazine and ISDN may provide a reasonable alternative to ACE inhibitors in patients who cannot tolerate ACE inhibitors or ARBs. More specifically, African‐American populations, in whom low‐renin, volume‐expansive hypertension is a common comorbid cardiovascular condition (Cook, 1988), the use of augmenters of ACE‐inhibitor action is encouraged to reduce the very high doses of ACE inhibitors otherwise required. The African‐American Heart Failure Trial randomly assigned 1050 Black patients with NYHA III/IV HF on standard medical therapy (including ACE inhibitors) to hydralazine and ISDN versus placebo. There was a significant decrease in all‐cause mortality in the treatment arm (*P* = 0.02) at 3 years (Taylor *et al*., 2004). Despite the compelling data for the use of organic nitrates in HFrEF, the use of nitrates in HFpEF has been relatively untested until recently, with many clinicians prescribing them to increase exercise tolerance and activity levels. The Nitrate's Effect on Activity Tolerance in Heart Failure trial, a multicentre, double‐blind crossover study of isosorbide mononitrate in HFpEF, demonstrated that, in fact, patients in the treatment arm trended towards being less active and had no beneficial increase in exercise capacity compared with placebo (Redfield *et al*., 2015).

### Cardiac glycosides

Positive inotropic effects combined with vagally mediated negatively chronotropic effects present a favourable profile for the use of cardiac glycosides. Outside of their use in managing tachyarrhythmias however, there has been no convincing effect on cardiovascular mortality in HF demonstrated. The role of digoxin in HF remained heatedly controversial given the risks for toxicity, despite over 200 years of clinical experience, until more recently, and the argument has been left largely unresolved. The largest study to look at the use of digoxin in HF patients was the Digitalis Investigation Group trial in 1997. This multicentre double‐blind trial assessed digoxin versus placebo in 6800 patients with a LVEF ≤45% on conventional medical therapy including diuretics and ACE inhibitors. All‐cause mortality was not significantly affected; however, fewer patients were hospitalized for worsening HF with a risk reduction of 28% (95% CI 21–34%, *P* < 0.001) (The Digitalis Investigation Group, 1997). A *post hoc* analysis of the Digitalis Investigation Group trial demonstrated a correlation between serum digoxin concentrations and crude all‐cause mortality, with significantly higher mortality in the toxic range and no benefit outside of the target range of 0.5 to 0.8 ng⋅mL^−1^ (Rathore *et al*., 2003), stressing the importance of regular plasma monitoring. A randomized, placebo‐controlled trial investigating the addition of digoxin to conventional therapy is ongoing (EudraCT:2007‐006660‐30).

### Ivabradine

A resting heart rate ≥70 bpm in patients with HFrEF has been shown to have a negative impact on cardiovascular outcomes (Fox *et al*., 2008). Ivabradine is a selective I*f* current inhibitor in the sinoatrial node, causing exclusively negative chronotropic effects. Ivabradine has therefore also found a role in the clinical setting for patients in whom β‐blockade is not tolerated or is contraindicated. The morbidity‐mortality evaluation of the I*f* inhibitor ivabradine in patients with coronary disease and left ventricular dysfunction trial (BEAUTIFUL; 2008) and systolic heart failure treatment with the I*f* inhibitor ivabradine trial (SHIFT; 2010) studies were pivotal trials underpinning the evidence for ivabradine in HF. The BEAUTIFUL trial assessed 10 917 HFrEF patients with stable coronary artery disease and a resting heart rate ≥60 bpm in sinus rhythm despite maximally tolerated β‐blockade. This trial failed to demonstrate a reduction in mortality, but suggested a trend towards benefit in patients with resting HR ≥70 bpm (Fox *et al*., 2008). In 2010, The SHIFT trial randomized 6558 patients with HFrEF who were in sinus rhythm with HR ≥70 bpm and had been admitted to hospital for HF within the previous year to receive ivabradine or placebo. Ivabradine reduced both the risk of death due to HF compared with placebo and hospital admissions for worsening HF by 26% (*P* < 0.0001) (Swedberg *et al*., 2010). These promising data have been challenged however, as despite 90% of patients receiving β‐blockers, only 26% of patients were successfully titrated to full doses, and up to 40% of patients were not managed with a mineralocorticoid receptor antagonist, suggesting sub‐optimal concomitant conventional medical therapy (Teerlink, 2010). Indeed, in a recent and much larger study of 19 102 patients with stable coronary artery disease without clinical evidence of HF, targeted heart rate reduction to a mean of 61 bpm conferred no demonstrable benefit in the treatment arm, and in fact, a conflicting increase was observed in the composite primary endpoint of cardiovascular death and non‐fatal acute myocardial infarction in patients with severe activity‐limiting angina (Fox *et al*., 2014). The debate on the role of ivabradine in cardiovascular disease is therefore ongoing.

### Neprilysin inhibition

Heart failure stimulates the compensatory natriuretic peptide system, which is composed of atrial natriuretic peptide, brain natriuretic peptide (BNP), C‐type natriuretic peptide and other vasoactive substances such as angiotensin II and bradykinin (Vardeny *et al*., 2014). Neprilysin degrades these vasoactive peptides, and so targeted inhibitors of neprilysin increase circulating levels of these substances and counteract the effects of neurohormonal over‐activation. Sole inhibitors of neprilysin, however, failed to demonstrate a significant impact on blood pressure in hypertensive cohorts and so were discontinued (McDowell and Nicholls, 1999). This may have been in part due to an attendant increase in angiotensin, necessitating the combination of neprilysin inhibitors with drugs that target the RAAS. Omapatrilat, the first dual neprilysin and ACE inhibitor, was shown to be superior to enalapril alone (Kostis *et al*., 2004). Unfortunately, there was a significant increase in the incidence of angioedema in the treatment arm (likely to be due to the substantial inhibition of bradykinin degradation), and the drug was discontinued. LCZ696, a combination of sacubitril (neprilysin inhibitor) and valsartan (ARB), as previously stated, showed a similar reduction in all‐cause mortality compared with an ACE inhibitor combination, with better safety data due to an attenuated effect on bradykinin breakdown (Packer *et al*., 2015).

## Metabolic dysfunction in heart failure

### Normal cardiac metabolism

The continuous and coordinated contractile activity of cardiac myocytes requires the consumption of vast amounts of energy, and this is reflected in the observation of tremendous mitochondrial density on histology (Goffart *et al*., 2004). Fatty acid (FA) metabolism predominates in adult healthy cardiac myocytes (around 70%), which is complemented by carbohydrate oxidation (20%) and, to a lesser extent, contribution from ketones and various amino acids (Stanley *et al*., 2005). However, the healthy heart is a metabolic ‘omnivore’, which is able to adjust substrate utilization according to substrate availability (Singh *et al*., 2014). Glucose enters the myocyte through glucose transporters (predominantly GLUT4). Glucose also undergoes a series of metabolic conversions in the cytosol known as glycolysis, resulting in the production of pyruvate, which is actively transported across the mitochondrial membrane where the pyruvate dehydrogenase (PDH) enzyme complex catalyses its conversion to acetyl CoA. PDH is the rate‐limiting step in carbohydrate metabolism and is negatively regulated by kinases and positively regulated by phosphatases (Doenst *et al*., 2013). Hypoxia potently inhibits PDH; therefore in these circumstances pyruvate is converted to lactate (anaerobic glycolysis). PDH is also subject to marked allosteric regulation, with acetyl CoA and NAD^+^ inhibiting PDH via kinase activation.

**Figure 2:**
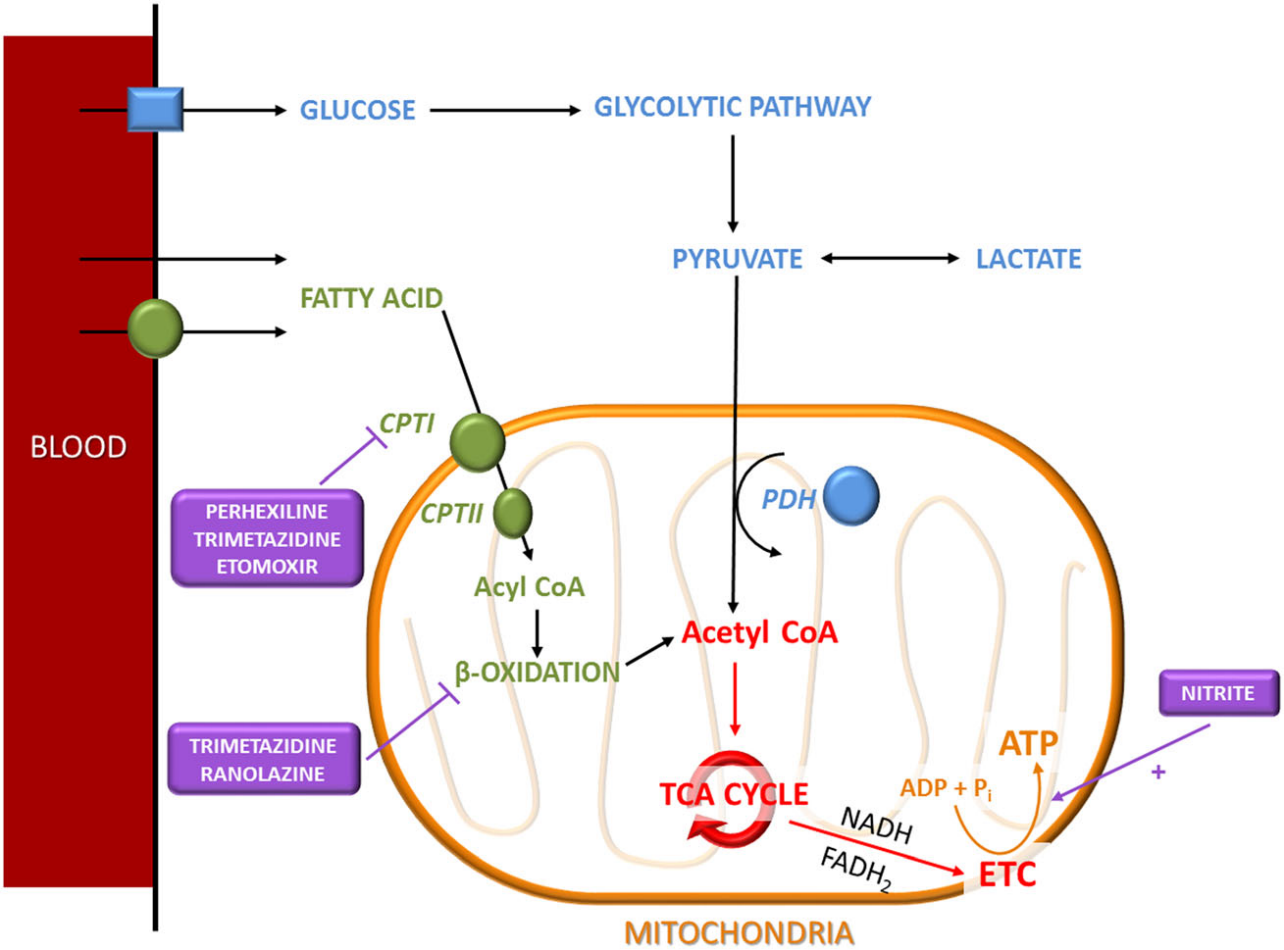
Schematic of the key metabolic pathways in cardiac myocytes. Fatty acid and glucose metabolism are key metabolic pathways within cardiac myocytes that are responsible for generating large amounts of ATP. Perhexiline and nitrite are therapeutic agents that have the ability to modulate and enhance cardiac metabolism. Acetyl CoA, acetyl coenzyme A; ADP, adenosine diphosphate; ATP, adenosine triphosphate; CPT, carnitine palmitoyltransferase; ETC, electron transport chain; FADH_2_, flavin adenine dinucleotide; NADH, nicotine adenine dinucleotide; P_i_, inorganic phosphate; TCA, tricarboxylic acid.

Long‐chain fatty acids enter the myocyte via fatty acid transporters and undergo esterification to long‐chain fatty acyl CoA. These are actively transported into the mitochondria via the ‘carnitine shuttle’ (van der Vusse *et al*., 2000). The enzyme carnitine palmitoyltransferase type 1 (CPT1) catalyses the addition of a carnitine group to the fatty acyl CoA molecule, facilitating transfer across the mitochondrial membrane where the enzyme CPT2 cleaves the carnitine (which is exported back across the mitochondrial membrane). In the mitochondria, the fatty acyl CoA undergoes β‐oxidation, a four‐step process generating acetyl CoA, NADH and flavin adenine dinucleotide (FADH_2_). Acetyl CoA arising from both fuel sources enters the tricarboxylic acid (TCA) cycle to produce NADH (Schwarz *et al*., 2014). High‐energy electrons donated from NADH and FADH_2_ (derived from succinate dehydrogenase, another component of the TCA cycle) are transferred from complex to complex of the electron transport chain to power extrusion of hydrogen ions across the membrane to generate an electrochemical gradient. This ultimately powers phosphorylation of ADP to ATP by ATP synthase within the mitochondrial inner membrane, with oxidation of the hydrogen ions to water (Figure 2). ATP within the mitochondria donates high‐energy phosphate to creatine catalysed by mitochondrial creatine kinase (CK), which is transported to the sites of energy utilization within the cytosol (Neubauer, 2007). Here, phosphocreatine (PCr) phosphorylates ADP to ATP (catalysed by cytosolic CK) to power the multitude of myocardial sarcomeres and various ion channels.

The high‐energy nature of the electrons utilized in oxidative phosphorylation results in the formation of harmful reactive oxygen species (ROS) (Schwarz *et al*., 2014), even in physiological states. Complex homeostatic mechanisms, including NADPH from the pentose phosphate pathway (PPP), exist to manage low levels of ROS and protect cardiac myocytes from detrimental mitochondrial DNA damage and subsequent activation of apoptotic pathways. Uncoupling proteins [both uncoupling protein (UCP) and adenine nucleotide translocase (ANT)] facilitate proton leak back into the mitochondria resulting in reduced ROS generation but also less ATP production. The activity of UCPs and ANT are increased by ROS (Schwarz *et al*., 2014).

### Metabolic changes in heart failure

Given the high‐energy consumption within the normal heart, it is appropriate to define HF as a condition in which metabolic impairment is a predominant feature. A decrease in ATP production (with resultant energetic starvation) resulting from impaired generation from glucose and FA and activation of energy‐consuming biosynthetic and redox‐stress pathways are mechanisms by which this metabolic shift occurs (Doenst *et al*., 2013). Creatine delivery and CK activity and flux are also reduced in HF (impairing ATP delivery to sarcomeres and ion channels necessary for pump function and cardiac homeostasis), which causes a reduction in the energy charge within the cytosol, as reduced delivery of ATP reduces the phosphorylation potential of ADP (given by Gibbs Law [ATP]/[ADP] [Pi]) (Siddiqi *et al*., 2013). These complex changes in the HF population are inconsistent across the aetiologies of HF, however, they become more uniform across groups at the onset of systolic dysfunction. A mechanistic study of 14 patients with aortic stenosis demonstrated a reduced PCr to ATP ratio on magnetic resonance spectroscopy in patients with symptoms of HF compared with those without (1.1 vs. 1.56, *P* < 0.0035) (Conway *et al*., 1991). PCr to ATP ratio has been well validated as a marker of cardiac metabolic status and efficiency (Beadle and Frenneaux, 2010).

The reduction in ATP production in HF is complex. Catecholaminergic stimulation in HF results in activation of lipolytic pathways and an increase in plasma‐free FAs. As a result, FA accumulation occurs within cardiac myocytes and mitochondria. Down‐regulation of enzymes for β‐oxidation occurs due to a decrease in activity of the PPARα pathway, which is a transcriptional regulator of FA use (Doenst *et al*., 2013). Decreased FA uptake and oxidation (with preferential utilization of carbohydrates) have been observed in patients with dilated cardiomyopathy and symptoms of HF compared with healthy controls (Neglia *et al*., 2007). In a further molecular study analysing human left ventricular biopsy tissue, total expression and mRNA levels of medium‐chain and long‐chain acyl CoA dehydrogenase (involved in FA β‐oxidation) were down‐regulated by >40% (*P* < 0.05) in patients with HF compared with age‐matched controls (Sack *et al*., 1996). A ‘funnelling’ effect occurs due to reduced β‐oxidation and increased mitochondrial FA in HF, and FAs begin to react with ROS within mitochondria to form lipid peroxides. Lipid peroxides damage mitochondrial DNA (hence impairing their ATP‐production capacity) and other cellular structures and activate MAPK pathways, triggering adverse remodelling and fibrosis (Siddiqi *et al*., 2013). Lipid peroxides also increase the activation of UCPs and ANT on the mitochondrial membrane, a protective mechanism allowing the mitochondrion to transport them back into the cytosol (Schrauwen and Hesselink, 2004). Unfortunately, this allows for proton leak at the electron transport chain and reduces the efficiency of ATP production.

Glucose oxidation is also impaired in patients with HFrEF. There is profound cardiac insulin resistance in HF, with decreased insulin‐dependent glucose uptake, but normal or increased insulin‐independent glucose uptake. G6P (formed by phosphorylation of glucose on entry into the cardiac myocyte) is also shunted down other metabolic pathways that do not produce ATP (Doenst *et al*., 2013). The hexosamine biosynthesis pathway, a growth and protein synthesis pathway, is increased in HF and contributes to cardiac hypertrophy and remodelling. The PPP is also up‐regulated to produce increased levels of NADPH, which are required for anti‐oxidative defence at low levels of ROS (Burgoyne *et al*., 2012), but may contribute to oxidative stress when ROS levels are high. G6P will also enter the glycolytic pathway to produce pyruvate. In HF, there is impaired oxidation of pyruvate to acetyl CoA by the PDH complex, reducing the amount available for entry into the TCA cycle. A high‐salt diet‐induced HF model in rats demonstrated a reduction in GLUT1 and PDH expression (Kato *et al*., 2010), and this was replicated in an *in vivo* thoracic aortic constriction HF murine model (Dai *et al*., 2013). PDH activity also appears to be reduced in HF resulting in impaired carbohydrate oxidation despite the availability of pyruvate (Singh *et al*., 2014). Pyruvate levels available for oxidation are also reduced in HF, as it is shunted along anaplerotic pathways (without ATP production) to replenish amino acids within the TCA cycle that have been utilized in hypertrophic growth and remodelling. The anaplerosis of pyruvate is enhanced in the presence of NADPH (derived from the up‐regulated PPP) and may consume large amounts of NADPH and impair anti‐oxidative defence (Doenst *et al*., 2013). The changes in cardiac metabolism culminate in hypertrophy and fibrosis reduced availability of ATP and high ROS levels, which further worsen the structural changes and energetic starvation.

### Metabolic modulators

Glucose metabolism is more efficient than FA metabolism (in terms of oxygen requirement), and therefore, shifting energy production towards this system (generally through direct or indirect PDH complex activation) in patients with cardiovascular disease has been suggested in order to correct metabolic impairment and reduce ROS production (Lopaschuk *et al*., 2002). Various metabolic modulators exist, including perhexiline, trimetazidine, ranolazine and etomoxir. Of these agents, perhexiline has shown great promise due to its high potency and well‐documented anti‐ischaemic properties (Horowitz and Chirkov, 2010).

Perhexiline was widely used throughout the 1970s for the treatment of refractory angina pectoris (Ashrafian *et al*., 2007). It was later revealed that perhexiline improved cardiac function and myocardial efficiency by down‐regulating CPT1 activity, the rate‐limiting enzyme required for transport of FAs across the mitochondrial membrane (Kennedy *et al*., 1996). This in turn prevents mitochondrial FA build up, limiting harmful ROS production and subsequent energetic impairment. Recently, it has also been proposed that perhexiline may directly increase activity of the PDH complex (Yin *et al*., 2013). This improved glucose metabolism at low oxygen tensions improves metabolic efficiency and, when used at earlier stages of cardiovascular disease, may slow progression. Despite these promising findings, the use of perhexiline drastically declined during the 1980s due to several adverse effects. These ranged from minor nausea and lethargy to debilitating peripheral neuropathy and hepatotoxicity (Shah *et al*., 1982). Severe toxicity, however, results from direct inhibition of liver and brain isoforms of the CPT1 enzyme, which may be avoided by tightly monitoring plasma concentrations within a target therapeutic range of 0.5–2.2 μM (Horowitz *et al*., 1986).

In a cohort study involving elderly patients aged >75 years with inoperable aortic stenosis, treatment with perhexiline improved NYHA functional class (*P* < 0.01), with an 80% 12‐month actuarial survival rate (Unger *et al*., 1997). In an RCT performed by our group involving 46 patients with hypertrophic cardiomyopathy treated with perhexiline or placebo for a mean of 4.6 months, improved cardiac energetics were demonstrated by improved PCr/ATP ratio (1.27 to 1.73, *P* = 0.03) and NYHA class (*P* < 0.001) in the perhexiline arm (Abozguia *et al*., 2010). A further RCT by us focusing on patients with HFrEF has also shown significant improvements in peak exercise oxygen consumption (16.1 ± 0.6 vs. 18.8 ± 1.1 mL⋅kg^−1^⋅min^−1^, *P* < 0.001) and LVEF (24% vs. 34%, *P* < 0.001) following treatment with perhexiline (Lee *et al*., 2005).

Trimetazidine is a weak CPT1 inhibitor (Kennedy and Horowitz, 1998) with potent inhibitory effects on the long‐chain 3‐ketoacyl‐CoA thiolase enzyme, which is involved in the final steps of FA β‐oxidation (Kantor *et al*., 2000). Similarly to perhexiline, trimetazidine is an effective antianginal and has been shown to increase time to exercise tolerance in combination with metoprolol (420 ± 108 s in the placebo group to 485 ± 122 s in the treatment arm, *P* < 0.05) in patients with chronic stable angina (Trimetazidine in Poland II study trial) (Szwed *et al*., 2001). Trimetazidine was further shown to improve left ventricular end‐systolic volume (98 ± 1.36 mL vs. 81 ± 27 mL, *P* = 0.04), with a corresponding increase in ejection fraction (36 ± 7% vs. 43 ± 10%, *P* = 0.002) (Fragasso *et al*., 2006).

Ranolazine has also been found to have antianginal effects and holds a similar chemical structure to trimetazidine (Horowitz and Chirkov, 2010). The exact mechanism of action remains unknown; however, in a global ischaemia rat model, ranolazine was found to partially inhibit FA oxidation and stimulate glucose oxidation (McCormack *et al*., 1996). These metabolic changes were shown to have therapeutic effects in a three‐group (placebo vs. one of two doses of ranolazine), parallel, double‐blind placebo‐controlled trial involving 823 chronic stable angina sufferers on background antianginal therapy (Combination Assessment of Ranolazine in Stable Angina Study trial). The treatment groups experienced an increased time to angina on exercise and could exercise for longer compared with the placebo arm (Chaitman *et al*., 2004). Nitroglycerin use also decreased in the treatment arm by one use per week vs. placebo (*P* < 0.02). In a recent, small proof‐of‐concept RCT, ranolazine was further shown to improve haemodynamics [with decreases in left ventricular end‐diastolic pressure (*P* = 0.04) and pulmonary capillary wedge pressure (*P* = 0.04) in patients with HFpEF (Maier *et al*., 2013)].

Etomoxir also inhibits the CPT1 enzyme, although to a lesser extent than perhexiline (Luiken *et al*., 2009). In a failing rat‐heart model, perfusion with etomoxir improved left ventricular haemodynamics and indices of myocardial performance (*P* < 0.05) (Turcani and Rupp, 1997). A small first‐in‐human study of etomoxir in 10 HF patients of non‐ischaemic origin demonstrated haemodynamic improvement in HFrEF, with an increase in LVEF (21.5 ± 2.6% to 27.0 ± 2.3%, *P* < 0.01) and exercise cardiac output (from 9.72 ± 1.25 L⋅min^−1^ to 13.44 ± 1.50 L⋅min^−1^, *P* < 0.01) (Schmidt‐Schweda and Holubarsch, 2000). The long‐term safety of etomoxir, however, has been questioned due to a recent clinical trial involving 260 patients with moderate HF, which was halted early due to four patients developing significant hepatic transaminitis (Holubarsch *et al*., 2007).

## A novel therapeutic pathway: nitrate–nitrite–NO

The nitrate–nitrite–NO pathway has been suggested as an alternative mechanism for systemic NO production. This is an alternative mechanism to the classical pathway in which NO is produced by oxidation of L‐arginine in a reaction catalysed by nitric oxide synthase (NOS) (Lundberg *et al*., 2008). NO generated through the nitrate–nitrite–NO pathway has been suggested to involve a series of nitric oxide synthase (NOS)‐independent and oxygen‐independent reactions. However, the precise mechanism(s) for nitrite conversion to NO remains to be fully elucidated because a number of enzymes have been implicated in the catalysis of nitrite to NO in various tissue compartments (Lundberg *et al*., 2008). Green leafy vegetables (such as spinach and beetroot) are the major source of dietary inorganic nitrate. Once consumed, the salivary inorganic nitrate is reduced to nitrite by commensal bacteria. Nitrite is then swallowed, absorbed from the stomach and reduced to NO, particularly under hypoxic conditions, by a number of nitrite reductases such as myoglobin, haemoglobin and xanthine oxidoreductase (Gladwin and Kim‐Shapiro, 2008; Webb *et al*., 2008). NO may then directly modify proteins [particularly via S‐nitrosylation of cysteine residues, which may enhance or dampen protein activity (Foster *et al*., 2003)] or, more classically, activate soluble guanylate cyclase within vascular smooth muscle cells and platelets to increase cGMP to exert its vasodilator and platelet‐inhibitory effects. Phosphodiesterase is pivotal in homeostatic control over cellular cGMP and cAMP activity; thus, targeted inhibition of cardiac and smooth muscle isoforms of phosphodiesterase have been shown to elicit vasodilatation and attenuate cardiac hypertrophy (Guazzi, 2008). A placebo‐controlled RCT involving 23 stable HF patients treated with the PDE5 inhibitor sildenafil for 6 months demonstrated an improvement in pulmonary artery systolic pressure (33.7 to 23.9 mmHg, *P* < 0.01) and aerobic efficiency and exercise ventilation (peak VO2 14.8 to 18.7 mL⋅min^−1^⋅kg^−1^, *P* < 0.01) (Guazzi *et al*., 2007). These promising data are yet to be explored in larger clinical trials.

Exploitation of the NO pathway has been further attempted with other stimulators and activators of soluble guanylate cyclase including riociguat, vericiguat and cinaciguat. Recently, the Soluble Guanylate Cyclase Stimulator in Heart Failure Study (in patients with LVEF <45%) trial, a randomized‐controlled, multicentre double‐blind trial to assess the tolerability and optimal dose of vericiguat in 456 patients with HFrEF, confirmed tolerability but failed to show a significant decrease in NT‐proBNP in the treatment groups (the primary endpoint). There was, however, a demonstrable dose–response relationship for the reduction in NT‐proBNP (*P* < 0.02), which was promising and requires further elucidation (Gheorghiade *et al*., 2015).

The nitrate–nitrite–NO pathway has been shown to regulate blood pressure and hypoxic vasodilatation and improve exercise performance and mitochondrial efficiency (Bailey *et al*., 2014). Our work and those of others have demonstrated that infusions of sodium nitrite in healthy subjects dilate forearm vasculature, with significant potentiation during hypoxia, thereby preferentially dilating capacitance vessels (Maher *et al*., 2008; van Faassen *et al*., 2009; Totzeck *et al*., 2012; Kapil *et al*., 2014). As such, these haemodynamic effects have sparked interest in inorganic nitrate (to increase serum nitrite) as a novel oral therapeutic intervention, particularly in cardiovascular diseases such as HFrEF. We have recently shown in a first‐in‐human HF efficacy/safety study that sodium nitrite infusion (50 μg⋅kg^−1^⋅min^−1^) in patients with severe HF resulted in an increase in ventricular stroke volume (43.22 ± 21.5 to 51.84 ± 23.6 mL, *P* = 0.003), with marked falls in pulmonary vascular resistance (29%; *P* = 0.03) and right atrial pressure (40%; *P* = 0.007), but only modest falls in mean arterial blood pressure (4 mm Hg; *P* = 0.004). The increase in stroke volume correlated (*r* = 0.67; *P* = 0.003) with an increase in estimated trans‐septal gradient (= pulmonary capillary wedge pressure − right atrial pressure), suggesting relief of diastolic ventricular interaction as a contributory mechanism (Ormerod *et al*., 2015).

Nitrite has also been shown to improve the metabolic efficiency of skeletal muscle. This may be partially due to its vasodilator properties, but it has also been shown to have direct effects on key metabolic components (Clerc *et al*., 2007; Basu *et al*., 2008). The Karolinska group showed in a double‐blind crossover trial in healthy volunteers that oral inorganic nitrate supplementation improves oxidative phosphorylation efficiency in skeletal muscle mitochondria. An analysis of mitochondrial function revealed decreased expression of ANT and decreased mitochondrial membrane uncoupling (Larsen *et al*., 2011). It has also been proposed that under hypoxic conditions, nitrite may act as the terminal electron acceptor in place of oxygen (Basu *et al*., 2008). Given the underlying metabolic impairment in HF and the potential benefit of metabolic modulation, determining whether changes observed in skeletal muscle translates to cardiac muscle would be of great benefit, but remains yet unclear. Zamani *et al*. (2015) reported in a double‐blind, placebo‐controlled crossover study that nitrate rich beetroot juice (12.9 mmol) in patients with HFpEF increased exercise cardiac output and improved peak VO_2_ compared with nitrate‐deplete placebo (Zamani *et al*., 2015). These new studies provide evidence of promising beneficial effects in patients with HF, and phase 2 studies for longer treatment regimens are underway (ISRCTN16356908; NCT02256345; ACTRN12615000906550; ACTRN12613000689774; NCT02401126).

## Conclusion

Classic treatments for HF have resulted in significant improvements in disease morbidity and mortality over the last 20 years. Despite this, cohorts of patients remain who cannot tolerate maximal up‐titration of traditional treatment modalities or remain symptomatic despite them. With rapid improvements in cardiac devices, we may be entering the surgical age of HF treatment, but such interventions remain expensive and inaccessible to many. Novel pharmacotherapeutic agents, such as perhexiline and nitrite, may provide pharmacological alternatives to traditional treatments and fill the therapeutic gap, but further definitive trials are warranted.

## Author contributions

B.L.L., H.N. and N.D.G. contributed to writing the article. M.P.F. and M.M. contributed to article drafting and revision. M.M. authorized the final version of the review.

## Conflict of interest

M.P.F. is the inventor for a method‐of‐use patent for the use of perhexiline in heart failure and cardiomyopathies. B.L.L., H.N., N.D.G. and M.M. have no conflict of interest to report.
